# Biomechanical, Biochemical, and Cell Biological Evaluation of Different Collagen Scaffolds for Tendon Augmentation

**DOI:** 10.1155/2018/7246716

**Published:** 2018-05-09

**Authors:** Carolin Gabler, Juliane Spohn, Thomas Tischer, Rainer Bader

**Affiliations:** ^1^Biomechanics and Implant Technology Research Laboratory, Department of Orthopedics, University Medicine Rostock, Doberaner Strasse 142, 18057 Rostock, Germany; ^2^Fraunhofer Institute for Ceramic Technologies and Systems IKTS, Perlickstrasse 1, 04103 Leipzig, Germany

## Abstract

Tendon augmentation is increasingly clinically relevant due to rising amount of tendon ruptures because of the aging and more demanding population. Therefore, newly developed scaffolds based on bovine epoxide stabilized collagen maintaining the native fibril-like collagen structure were characterized and compared to two commercially available porcine collagen scaffolds. For biomechanical testing (ultimate load, ultimate stress, stiffness, and elastic modulus), bovine collagen scaffolds were hydrated and compared to reference products. Cell viability and proliferation were assessed by seeding human primary fibroblasts on each collagen-based scaffold and cultured over various time periods (3 d, 7 d, and 14 d). Live/dead staining was performed and metabolic cell activity (WST-1 assay) was measured. Biochemical degradability was investigated by enzymatic digestion. The bovine collagen scaffold showed significantly enhanced biomechanical properties. These persisted over different rehydration times. Cell biological tests revealed that the bovine collagen scaffolds support reproducible cell colonization and a significant increase in the number of viable cells during cultivation. The results are comparable with the viability and proliferation rate of cells grown on porcine reference materials. With regard to biochemical degradability, all tested materials showed comparable resistance to enzymatic degradation* in vitro*. Due to imitating the natural tendon structure the new scaffold material is supposed to provide beneficial effects in future clinical application.

## 1. Introduction

Tendon disorders are frequent and usually cause high morbidity. For example, the rotator cuff, anterior cruciate ligament, and Achilles tendon are susceptible to injuries due to occupational or sporting activities. In addition, elderly people suffer tendon lesions due to degenerative changes in the tendon caused by biological and mechanical reasons, leading to an increased risk of rupture. For example, it is reported that rotator cuff tears affect at least 40% of patients aged 60 years and more [1]. Pain and functional loss often require surgical treatment. In Germany, nearly 50,000 inpatient treatments are performed due to lesions of the rotator cuff per year [2]. In the USA up to 300,000 rotator cuff repairs are performed annually [3, 4].

Despite improvements in surgical treatment, healing after rotator cuff or other tendon repairs often remains a clinical challenge. A mean repair failure rate of 26.6% has been reported [5], but the rate can go up as high as 90% [6] depending on factors such as the patient's age, tear size, tendon quality, degeneration, repair technique, and postoperative rehabilitation [1, 3]. Therefore, tendon augmentation with scaffolds could provide a more effective management option with increased healing rates. In order to be successful, these scaffolds should provide biomechanical support and improve the tendon healing process [7–9].

Several synthetic and biological biomaterials are available for the repair and augmentation of large tendon defects. Synthetic scaffolds contain chemical compounds to control chemical and physical properties of the product within the manufacturing process [10]. Hence, a higher initial mechanical strength of the scaffold and stable product characteristics can be obtained. However, synthetic scaffolds lack biocompatibility and long-term mechanical stability. Therefore, synovitis, osteolysis, and foreign body reaction have often been reported [10, 11]. Biological scaffolds are based on extracellular matrices that are derived from human or animal connective tissue [11]. They are characterized by their natural porosity and 3D surface protein microstructure. These features enable improved biological performance such as host cell attachment, migration, and integration compared to synthetic scaffolds. On the other hand, they are often limited by their mechanical properties, undefined degradation rate, and variation of biocompatibility depending on the source of raw material. Although they are lacking in mechanical strength, biological scaffolds—especially allografts—tend to demonstrate the best clinical results [9, 11, 12].

Hence, in the present study newly developed scaffolds based on bovine collagen were characterized. Biomechanical, biochemical, and cell biological investigations were carried out and compared to two commercially available scaffolds as reference material, that is, DX Reinforcement (Arthrex, Arthrex Inc., Naples, FL, USA) and Conexa™ 100 Reconstructive Tissue Matrix (Tornier, Tornier Inc., Edina, MN, USA), approved for clinical use in human tendon repair.

## 2. Materials and Methods

### 2.1. Scaffolds

Preparation of scaffolds was based on an industrial scale production process established for manufacture of medical devices. Bovine dermal tissue was subjected to chemical treatments with NaOH, H_2_O_2_, and HCl for purification (i.e., removal of noncollagenous proteins, fatty acids) of the dermal collagen and safety treatment (i.e., removal of cells, inactivation of viruses, etc.). The obtained purified dermal fibrillar collagen (consisting mainly of collagens type I, type III, and type V) was processed to a collagen matrix which ensures a longitudinal orientation of collagen fibrils within the matrix in order to mimic the natural tendon structure and mechanical properties. This predefined matrix structure was stabilized by freeze-drying. Processing parameters of previously mentioned production modules generate a pure fibrillar collagen matrix and avoid destruction of collagen fibrils thereby maintaining the authentic native fibrillar collagen structure of the initial dermal tissue. Freeze-dried matrix was further stabilized by chemical crosslinking to achieve optimal tear strength. Chemical crosslinking was performed by subjection of the freeze-dried matrix to an aqueous epoxide solution. The removal of potential free epoxide was carried out by successively washing the crosslinked collagen matrix with reverse osmosis (RO) water.

Two different scaffold manufacturing configurations were compared. Material 1 was exposed to a freeze-drying temperature within the range of 55–65°C and the concentration of aqueous epoxide solution was 0.19% (w/w). Material 2 was manufactured with a freeze-drying temperature within the range of 100–120°C and aqueous epoxide solution of 0.38% (w/w). The collagen samples were gamma-sterilized before testing. After hydration in saline solution, the test samples (scaffolds) showed thicknesses of 0.9 mm (material 1) and 1.3 mm (material 2).

As reference materials, two commercially available products were used: DX Reinforcement (Arthrex Inc., Naples, FL, USA; thickness: 1.4 mm) based on porcine dermal extracellular matrix and Conexa 100 Reconstructive Tissue Matrix (Tornier Inc., Edina, MN, USA, thickness: 1.2 mm) based on porcine skin. The manufacturing processes of both materials do not contain crosslinking. Both materials were delivered hydrated.

### 2.2. Cell Biological Tests with Primary Human Fibroblasts

#### 2.2.1. Cell Isolation and Cultivation

Primary human fibroblasts were isolated from leftover skin remnants of the eyelids of anonymous patients (*n* = 3) undergoing plastic surgery. The use of fibroblasts was approved by the Local Ethical Committee of the University of Rostock (registration number: A 2013-0092). The skin biopsy was cleaned of fat, cut into small squares (2-3 mm^2^), and directly transferred to a 6-well plate (epidermis upward) where it was dried for 20 min at room temperature (RT). DMEM (Dulbecco's Modified Eagle Medium) supplemented with 10% fetal calf serum, 1% amphotericin B, and 1% penicillin/streptomycin (all from Thermo Fisher Scientific, Waltham, MA, USA) was added and the biopsies were incubated in a humidified atmosphere at 37°C and 5% CO_2_ with a medium change every week. After three weeks, fibroblastic cells grown out of the skin biopsy reached around 80% confluence and were trypsinized with 1% trypsin/EDTA (Thermo Fisher Scientific) and cultured in a 125 cm^2^ culture flask at 37°C and 5% CO_2_. The medium was changed every two to three days. Cells were cultured over several passages (P). For all experiments, cryoconserved fibroblasts were used. After thawing, cells were centrifuged at 118 ×g for 10 minutes, transferred into 75 cm^2^ flasks (P7 or P8), and incubated as described before. In P8/P9, 5 × 10^4^ cells (per patch, 0.5 mm in diameter) were transferred onto the collagen-based scaffolds which were introduced above, cultivated in a 48-well plate (one patch per well). We used suspension cell plates to prevent the cells from growing on the plate rather than on the scaffold.

#### 2.2.2. Cell Viability and Metabolic Cell Activity

By means of field emission scanning electron microscopy (FESEM; Merlin, Carl Zeiss, Jena, Germany) the surface structure of all collagen scaffolds in the hydrogenated state was analyzed.

The cell viability was assessed with a LIVE/DEAD^©^ assay kit (Thermo Fisher Scientific). The two-color assay discriminates vital from dead cells by simultaneously staining with green fluorescent (494–517 nm) calcein-acetoxymethyl (calcein-AM) to indicate intracellular esterase activity and red fluorescent (528–617 nm) ethidium homodimer-1 to predict the loss of plasma membrane integrity. The assay was performed as recommended by the manufacturer. Images of the cells were taken with a fluorescence microscope (Nikon Type 120; Nikon Corporation, Tokyo, Japan) and evaluated with NIS-Elements software (Nikon Corporation). Furthermore, scanning electron microscopy (SEM) with a DSM 960 A (Carl Zeiss Meditec AG, Jena, Germany) was performed to detect cell growth on the scaffolds.

The metabolic cell activity was analyzed using the colorimetric water-soluble-tetrazolium salt (WST-1) assay (Hoffmann-La Roche Ltd., Grenzach-Wyhlen, Germany) according to the manufacturer's instructions. In short, two die-cuts per material patch were transferred to a new one incubated with a mix of WST assay reagent and cell culture medium at a ratio of 1 to 10 for 60 minutes at 37°C. In metabolically active cells the tetrazolium salt WST is transformed to formazan by mitochondrial succinate dehydrogenase. The optical density (OD) was measured at 450 nm (reference 650 nm) using a Tecan reader (Infinite F200 Pro, Männedorf, Switzerland).

### 2.3. Enzymatic Digestion of the Scaffolds

The biochemical degradability was investigated by means of enzymatic digestion with pronase (protease type XIV; Sigma-Aldrich Chemie, Munich, Germany). Die-cuts (5 mm *∅*) were punched out from each material patch and dried at 50°C overnight. The die-cuts (three per material sample) were weighed to determine the mean initial dry weight. Sample die-cuts were than incubated in (1) RT (dry), (2) 37°C (dry), (3) NaCl/37°C, and (4) DMEM/37°C for seven days. After digestion with 1 U/mL pronase in Tris buffer for 24 h the reaction was stopped by adding an EDTA solution.

To determine whether human fibroblasts accelerate the enzymatic degradation of the scaffolds, we seeded 5 × 10^4^ cells (per patch, 0.5 mm *∅*), as described above, and incubated the patches for seven days and 21 days (*n* = 3). We used only 0.25 U/ml of pronase (instead of 1 U/ml) to make sure that we can detect and measure the cellular influence on the degradation velocity of fibroblasts on the enzymatic degradation of the scaffolds. After digestion with 0.25 U/mL pronase in Tris buffer for 24 h, the reaction was stopped by adding an EDTA solution. Control groups were incubated in Tris buffer only. After several washing steps and redrying, the die-cuts or stamped product residues were weighed again to determine the dry weight after digestion.

### 2.4. Biomechanical Testing

All test samples of test material configurations 1 and 2 were hydrated in saline solution for 30 min at RT. For each test material four samples were used. Reference materials were delivered hydrated. Five samples from two scaffolds from DX Reinforcement and three samples from one scaffold from Conexa 100 Reconstructive Tissue Matrix were evaluated biomechanically.

Hydrated test samples were cut into strips (2 cm × 5 cm) and fixed by means of a materials testing machine (Z1.0, Zwick, Ulm, Germany). A preload of 5 N was applied, followed by cyclic loading of between 5 and 50 N for 30 cycles and a final destructive test. The whole test procedure was measured at a distraction rate of 12.5 mm/s as described by Barber and Aziz-Jacobo [13]. Ultimate load (Fmax), ultimate stress (Rm), stiffness (S), and elastic modulus (EM) were evaluated.

To check for secure stable mechanical properties of the materials prepared by two different manufacturing configurations, the influence of dry storage at 40°C on the biomechanical properties was tested. Four scaffolds each were stored for one, three, and six months and compared to four scaffolds that were not stored. Samples were rehydrated in saline solution (0.9% NaCl, B. Braun Melsungen AG, Hessen, Germany) for 30 min at RT before testing.

To investigate the influence of temperature and the duration of rehydration on biomechanical properties, in total 36 test samples of both materials (1 and 2) were hydrated in saline solution at RT and 37°C, respectively, over various time periods (30 min, 24 h, 3 d, 7 d, and 14 d).

Furthermore, four test material samples of material 1 were hydrated for each of the time points (30 min, 24 h, 3 d, 7 d, and 14 d) in saline solution and Dulbecco's Modified Eagle's Medium (DMEM, Life Technologies GmbH, Darmstadt, Germany) at 37°C.

### 2.5. Statistical Analysis

Data presented in this work are shown in figures as mean ± standard deviation. The statistical analysis was performed using SPSS Statistics 20 (IBM Corp., Armonk, NY, USA). Pair-wise comparisons within the independent groups were performed using Student's* t*-test. *p* values less than 0.05 were considered as statistically significant.

## 3. Results

### 3.1. Cell Viability and Metabolic Cell Activity

**Figure 1:**
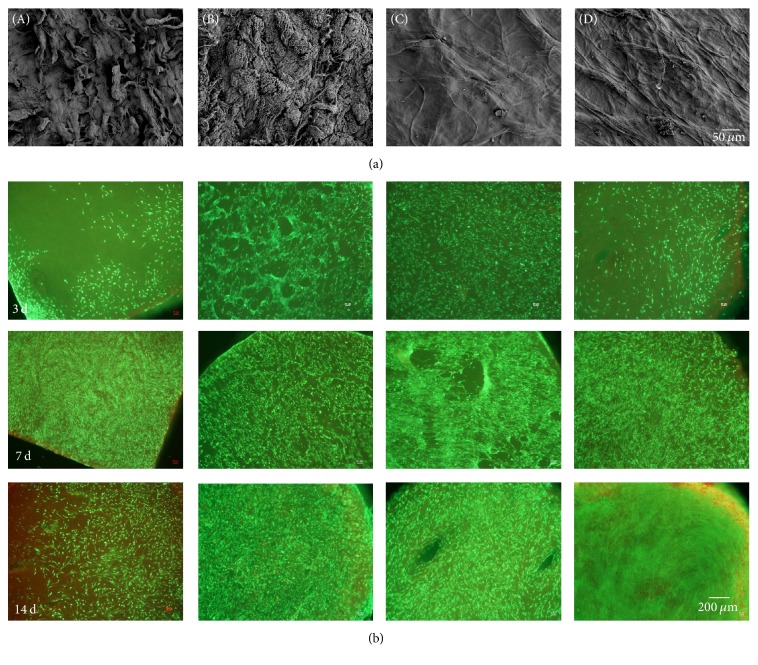
*Surface characterization of the collagen scaffolds and analysis of cell viability.* (a) Representative FESEM pictures showing the surface structure of the different hydrogenated scaffolds (without cells). (b) Live/dead staining of primary human fibroblasts cultivated for 3, 7, and 14 days on the collagen scaffolds (green = vital cells; red = dead cells). (A) DX Reinforcement, (B) Conexa 100, (C) Test Material 1, and (D) Test Material 2.

The FESEM analysis demonstrated that the surface structure of the hydrogenated test materials differs from that of the reference materials, as it appears much smoother altogether. The collagen fiber bundles are clearly visible and show a directed grid-like arrangement, while for both reference materials the collagen fibers seem to be disordered or very relaxed and fissured with high porosity (Figure 1(a)).

Primary human fibroblasts were seeded onto the different collagen scaffolds and live/dead staining was conducted (Figure 1(b)) after three, seven, and 14 days of cultivation.

There was a time-dependent increase in cell number on all scaffolds, except for DX Reinforcement scaffold. There we found less green fluorescent (vital) cells on the surface of the scaffold after 14 days of cultivation compared to day seven. On the Conexa 100 scaffold we found a higher percentage of dead cells, especially in the marginal zone.

**Figure 2:**
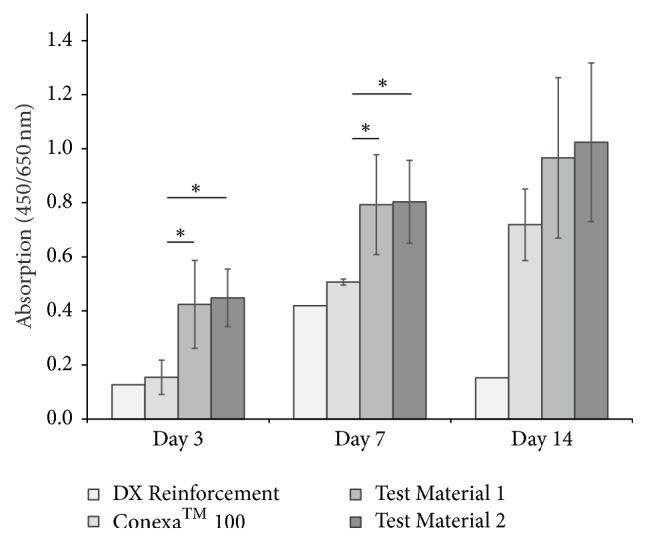
*Metabolic cell activity on different collagen scaffolds.* Primary human fibroblasts were cultivated on the bovine and porcine collagen scaffolds for 3, 7, and 14 days. Metabolic activity was analyzed via WST-1 assay. ^*∗*^*p* < 0.05 compared to Conexa 100.

Additionally, we analyzed the metabolic cell activity (Figure 2) and found an initially increased activity on both test materials compared to both reference materials after three and seven days of cultivation. The reduced cell activity on the DX Reinforcement scaffold points to increased cell infiltration, confirming the cell vitality results. Except for the DX Reinforcement material, we found a time-dependent cell proliferation consistent with the live/dead staining results.

### 3.2. Enzymatic Digestion of the Tendon Scaffolds

**Figure 3:**
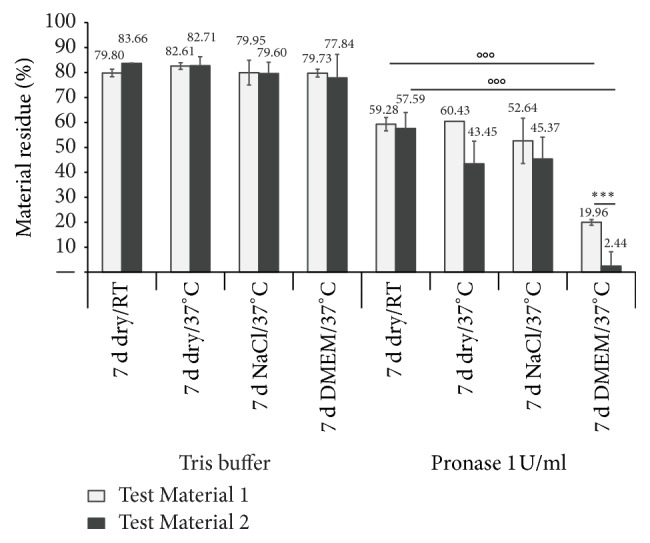
*Enzymatic degradability after 7 days of incubation in NaCl and cell culture medium (DMEM).* The material die-cuts and die-cut residues, respectively, were weighed before and after pronase digestion (incubation with 1 U/ml for 24 h) to determine changes in weight compared to the same material treated with Tris buffer (as positive control). ^ooo^*p* < 0.001 compared to 7 d dry/RT; ^*∗∗∗*^*p* < 0.001 compared between the two bovine test materials.

First, we incubated the scaffold patches under different conditions over seven days—(1) RT (dry), (2) 37°C (dry), (3) NaCl/37°C, and (4) DMEM/37°C—to see the extent to which the cell culture medium alone accelerates the enzymatic degradation process* in vitro* (Figure 3). In general, Test Material 2 was found to tend to degrade faster than Test Material 1. Additionally, we found a significantly reduced material residue after the incubation in DMEM for both bovine test material configurations compared to the control. Furthermore, we identified a significant difference between Test Material 1 (residue 19.96%) and Test Material 2 (residue only 2.44%).

**Figure 4:**
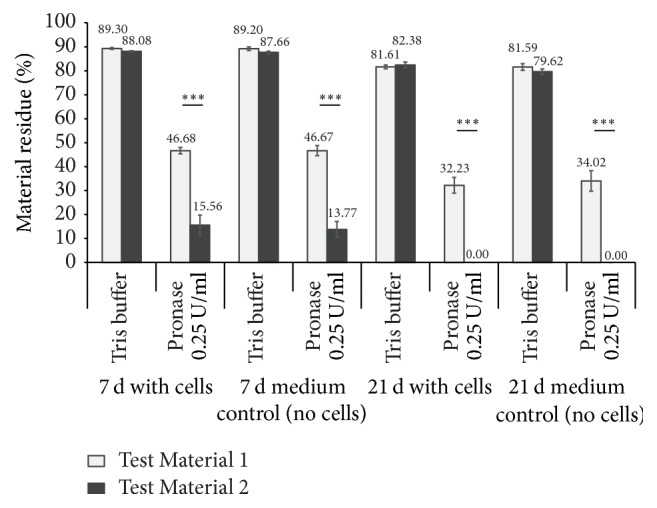
*Cellular impact on enzymatic degradation after 7 and 21 days of cultivation.* Primary human fibroblasts were cultivated on the two different bovine collagen test materials for 7 and 21 days. The stamped product residues were weighed before and after pronase digestion (0.25 U/ml) to determine changes in weight compared to the same material treated with Tris buffer (as positive control). ^*∗∗∗*^*p* < 0.001 compared between the two materials.

To investigate the possible stimulatory effect of fibroblasts on the enzymatic degradation of the scaffolds, we decided to use only 0.25 U/ml of pronase (instead of 1 U/ml) to ensure that we could measure the cellular influence on the degradation velocity. Primary human fibroblasts were seeded onto the collagen scaffold patches, as described above, and incubated for seven and 21 days (Figure 4).

We detected a significant time-dependent reduction in the material residues for both test materials. Test Material 2 had completely dissolved after 21 days of incubation in DMEM with and without cells. We could not detect an additional cellular impact on the degradation efficiency, either after seven or after 21 days.

### 3.3. Biomechanical Tests

#### 3.3.1. Evaluation of New Bovine Scaffolds

**Figure 5:**
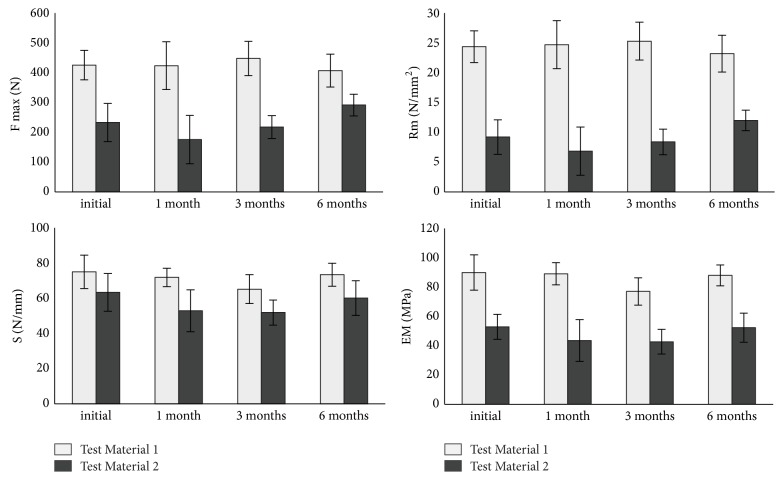
*Results of mechanical stability testing on bovine collagen scaffolds.* The test materials were stored at 40°C for up to six months. Ultimate load (Fmax), ultimate stress (Rm), stiffness (S), and elastic modulus (EM) were evaluated.

*(a) Mechanical Stability.* Both bovine test materials of the different manufacturing configurations showed an approximately stable biomechanical behavior over the storage periods of up to six months (see Figure 5). There is no negative influence of dry storage at 40°C on the materials. As described before, the biomechanical properties of Test Material 1 were higher than those of Material 2.

**Figure 6:**
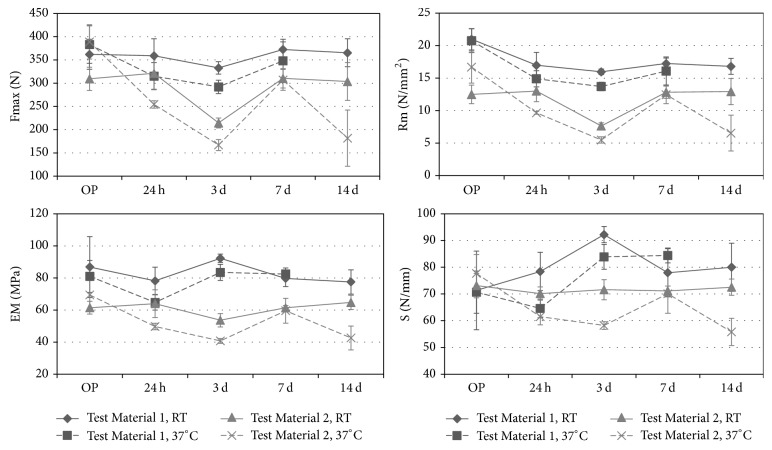
*Influence of temperature and duration of rehydration on biomechanical properties of bovine test materials.* Ultimate load (Fmax), ultimate stress (Rm), stiffness (S), and elastic modulus (EM) were evaluated.

*(b) Influence of Temperature and Duration of Rehydration on Biomechanical Properties. *The biomechanical properties of Test Material 1 nearly persisted over the time of rehydration (see Figure 6). Compared to the initial measurement after 30 min, Fmax remained almost unchanged up to 14 days of rehydration. For Rm a loss of about 19.9% and for EM a loss of about 10.7% after 14 days of rehydration were observed. There was a slight increase in stiffness of 12.1% after 14 days. The biomechanical properties at 37°C were observed only up to seven days. The rehydration at 37°C resulted in a decrease of Fmax (91%) and Rm (77.5%) compared to their initial values. The decrease is slightly stronger than the rehydration at room temperature after seven days. EM showed a decrease of 20% after 24 hours, but an increase after three days of rehydration at 37°C, returning to its initial value. The values for S decreased after 24 hours (91.4%) and increased after three days (3 d: 118.7%; 7 d: 119.4%).

The biomechanical properties of Test Material 2 remained almost unchanged over the period of rehydration at room temperature as well (Figure 6). Only at day three was a decrease of Fmax (69.3%), Rm (61.2%), and EM (87.3%) observed compared to the initial values. The rehydration at 37°C showed higher variations in biomechanical characteristics over the time and resulted in an overall clearer drop in the values after 14 days (Fmax: 46.7%; Rm: 39.2%; EM: 61.4%; S: 71.8%).

Overall, it can be stated that the period of rehydration at room temperature in 0.9% NaCl solution has little to no effect on the biomechanical characteristics, whereas the rehydration at 37°C in NaCl solution tends to result in a decrease of biomechanical values. All values of Test Material 1 remained higher than the initial values of reference materials.

**Figure 7:**
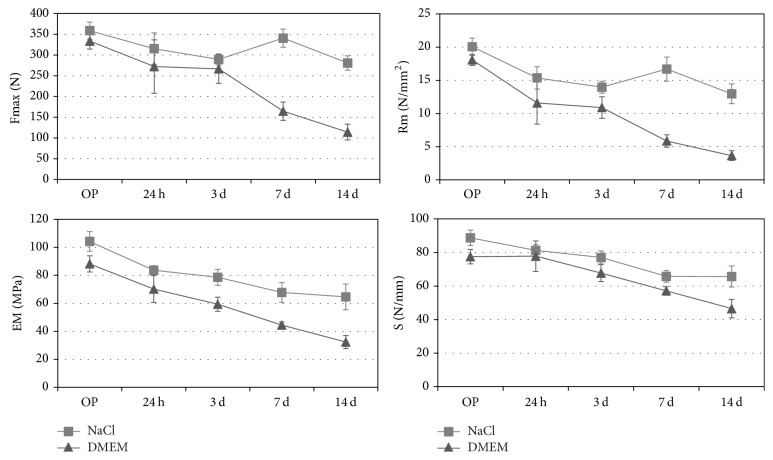
*Influence of medium during rehydration at 37*°*C of lab samples of bovine Test Material 1.* Ultimate load (Fmax), ultimate stress (Rm), stiffness (S), and elastic modulus (EM) were evaluated.

*(c) Influence of Medium during Rehydration at* 37°C. These investigations were only executed on Test Material 1 as this material showed higher and more constant biomechanical results than Material 2. Results were shown in Figure 7. The samples showed a moderate decrease of biomechanical values over the period of rehydration in 0.9% NaCl solution at 37°C. At day 14, values decreased to Fmax: 78.2%; Rm: 64.6%; EM: 62.0%; S: 74.0% compared to their initial values. Over the whole period investigated, the biomechanical values were higher than the initial values of both reference materials.

For all mechanical properties, the lowest values were found after rehydration at 37°C in DMEM for 14 days. Fmax decreased to 34.2%, Rm to 20.2%, and EM to 36.6% of their initial values. Only stiffness showed a moderate loss of 40.0%. Fmax and Rm were still higher than the initial values of reference materials up to three days of storage in DMEM at 37°C. EM of the test material was still higher after seven days compared to Conexa and after 14 days compared to DX Reinforcement. The stiffness of the test material was higher than both reference materials for the whole evaluation period.

#### 3.3.2. Comparison of Bovine Test Materials versus Porcine Reference Products

After 30 min of rehydration at RT both bovine collagen scaffolds (Test Materials 1 and 2) showed enhanced biomechanical properties compared to the porcine reference materials (see Table 1). There was a significant difference between the parameters of test materials in Rm (*p* < 0.001) and EM (*p* < 0.05), but not for Fmax and stiffness. The results showed that Test Material 1 tends to benefit in terms of the biomechanical behavior compared to Test Material 2 and the both reference products. The DX Reinforcement material showed significantly lower parameters than both test materials (*p* < 0.001). Compared to the Conexa 100 the DX Reinforcement scaffold differs only in stiffness (*p* < 0.05).

## 4. Discussion

Today approximately 30% of surgical tendon repairs of rotator cuff result in recurring tear [11]. In particular, the outcome of massive rotator cuff tear repair is less satisfactory and shows even higher failure rates [14–16]. In cases where the tendon defect is so large that it cannot be repaired with the use of native tissue, tendon augmentation with scaffolds can provide a more effective management option. Although biological scaffolds lack initial mechanical strength compared to synthetic materials, they have the inherent advantage of bioactivity. Thus, their natural 3D surface microstructure provides increased space for host cell attachment and enhances proliferation and migration. Human dermis grafts in particular were reported to be successful in clinical applications [9, 12, 17–20]. It was reported that the GraftJacket® scaffold (Wright Medical, Memphis, TN, USA) provides improved mechanical properties compared to other commercial products [13]. Steinhaus et al. [9] reported improvements in clinical and functional outcomes, with similar results for allografts and synthetic grafts, while xenografts were less effective. Also Ferguson et al. [12] stated that xenograft augmentation of large-to-massive rotator cuff repairs failed to demonstrate a superior outcome compared to synthetic grafts and conventional tendon healing. Therefore it should be noted that studies relating to xenografts include primarily small intestine submucosa (SIS) grafts, especially the Restore Patch (DePuy Orthopedic, Warsaw, IN, USA), which is known to cause complications in clinical use, as described by Chen et al. [11]. However, clinical outcomes showed also various results for synthetic grafts. The main complications in long-term studies included synovitis and foreign body reaction, which led to the fact that some grafts had already been removed from the market [11]. To overcome current limitations such as mechanical strength or lack of biocompatibility during the remodeling process the development of new scaffold materials is focused in biomedical research [21].

Therefore, in the present experimental study, a new scaffold material based on bovine collagen was analyzed* in vitro* and compared to commercially available reference materials. Bovine dermal collagen is particularly suitable as an extracellular matrix patch as it contains type I, III, and V collagens, similar to human tendons. It has to be mentioned that the usage of some graft materials is limited to some countries due to region-specific factors and missing approval of the product. In Germany, for example, allograft materials are subjected to regulations based on transplantation law. In Japan the use of allografts is not approved in general [22]. An additional advantage, besides the ease of availability of bovine collagen, is that it can also be used in patients who reject porcine products for religious reasons. The manufacturing process, especially the freeze-drying, ensures that a major part of the native collagen structure remains intact, a fact that is particularly important for the biocompatibility and cell colonization of the material. Longitudinal orientation of collagen fibers was ensured in order to mimic the natural tendon structure. The fabrication of aligned, mechanically strong collagen fiber scaffolds is expected to enhance their functionality by mimicking both the mechanical and biological tendon and ligament environment [23]. It has already been shown that fiber alignment functions as a topographical cue for cellular morphology and metabolism, for example, for an oriented deposition of collagen matrix [24–27]. In contrast to allografts or scaffolds based on ECM, the raw material undergoes preparatory processes for acellularization. Thus, the risk of a foreign body reaction to residual substances, as described, for example, for allogenic scaffolds, [28–30] is reduced. Biological scaffolds should offer a balanced degradability. Therefore, epoxide that is established and proven in medicine was used for mechanical stabilization and crosslinking of the insoluble collagen fibers. As crosslinking is known to be able to provoke adverse effects [31], removal of potential free epoxide was carried out due to a successive washing. All test samples were gamma-sterilized before testing to avoid unknown effects of the sterilization process on the material properties and to constitute a situation of clinical use.

As a limitation it has to be noted that the samples of the porcine commercially available reference materials were obtained from at most two patches at a time so that sample sizes were limited and no deviation between different batches of the test material was observed.

Our cell biological data showed that the number of vital fibroblastic cells increased on our test materials during cultivation. According to DIN ISO 10993-5, fibroblasts are generally used for* in vitro* analysis of biocompatibility properties and cytotoxic effects of biomaterials. Additionally, it was proven that the fibroblastic cell type is just as suitable for regeneration processes in tendon repair as tenocytes [32]. Our results show that the test materials support reproducible cell colonization (due to low standard deviations) and a significant increase in the number of viable cells during cultivation, especially from day three till day seven. Therefore, we conclude that the test materials are not cytotoxic.

In contrast, we found less green fluorescent cells on the reference product DX Reinforcement which might indicate that cells infiltrated this matrix between seven and 14 days of* in vitro* cultivation because of its more fissured surface structure with higher porosity compared to our test materials.

For the purpose of augmentation of tendon defects the ideal scaffold should not promote a fast cell infiltration to ensure a delayed biodegradability [33]. Thus stable initial mechanical properties of the scaffold could be maintained and fewer degradation products are produced resulting in higher biocompatibility.

Regarding our first preliminary test the fibroblasts seemed not to infiltrate our test materials within the first three weeks of cultivation* in vitro*. This was investigated by live/dead stained cross sections (data not shown) and by matrix degradation tests with pronase showing no increased degradation of the matrix when incubated with cells. We have cleaved the patches with a scalpel in the middle to recognize the infiltrated cells inside. Unfortunately, this manual procedure turned out to be difficult. On the one hand there were intersection artifacts and thus no evaluable sections. Furthermore, the microscopy of the sections was very difficult to carry out due to the small thickness diameter. We are planning to optimize the method for further investigations, for example, by using a laser to conduct contact-free cutting of the scaffold.

Our test method for enzymatic digestion, which we had to adjust to the scaffolds, was not exactly comparable to previous tests described in the literature based on MMPs [34]. But it might be a very promising test method for quantifying the matrix degradation* in vitro* whereby conclusions could be drawn about the* in vivo* situation. We used pronase which is very aggressive against collagen materials and thus simulates a very fast degradation, so to speak as a worst case scenario. As a physical support, the tendon patch should not break down so quickly. Therefore, a simulation of the worst case was applied and we used pronase in our assay.

The manufacturing process adjusted the biomechanical behavior of the test samples. Crosslinking was obtained due to thermal treatment under dehydration and chemical substances. In order to evaluate the optimal conditions, many preliminary test samples were manufactured and tested with different combinations of temperature during freeze-drying and concentration of the epoxide solution. The manufacturing processes of the reference materials do not contain crosslinking. However, source materials lead to a primary stabilization. Previously set material specifications were based on the characteristics of the human supraspinatus tendon. The investigation of the biomechanical behavior of the native human tendon, for example, the supraspinatus tendon of the rotator cuff, was beyond the scope of this study because, among other things, of the poor availability of fresh human tendons. Values in the literature are poor and vary widely as they use different test setups and protocols [35–38]. Both scaffold manufacturing configurations for the bovine collagen scaffold materials showed higher ultimate load, ultimate stress, stiffness, and elastic modulus than both reference materials used. Stability studies showed that the biomechanical behavior was stable over the storage periods of up to six months. The low standard deviations reveal that the manufacturing process provides reproducible results. Biomechanical characteristics of both materials were little or not time dependent during rehydration in saline solution at room temperature. The rehydration at 37°C tends to result in a decrease of ultimate load, stress, stiffness, and elastic modulus. Test Material 1* (55–65*°C* at freeze-drying, 0.19% (w/w) epoxide concentration for chemical crosslinking)* showed an improved mechanical behavior compared to Material 2* (100–120*°C* at freeze-drying, 0.38% (w/w) epoxide concentration for chemical crosslinking)* and remained higher over rehydration time than the initial values of the reference materials. The ultimate load and stiffness of Material 1 were comparable to the GraftJacket matrix tested by Barber and Aziz-Jacobo [13]. The rehydration in DMEM at 37°C resulted in a continuous decrease of all measured biomechanical values indicating an onset of material degradation. In a first trial we failed to observe any impact of fibroblasts of mechanical characteristics due to the reduced maximum loads after 21 days' incubation in DMEM. These scaffolds did not survive the precondition of testing protocol (data not shown). Therefore, another protocol with less or no precondition would need to be established. Further investigations have to be carried out to detect if human fibroblasts affect the biomechanical characteristics over time.

## 5. Conclusion

We analyzed a new scaffold material for augmentation of tendon tissue based on bovine collagen. The raw material underwent preparatory and preprocessing processes for acellularization, but the manufacturing process ensures that a major part of the native collagen structure remains intact. Acellularization must be ensured in order to minimize the risk of foreign body reaction to residual substances.

Our experimental data demonstrate that the bovine scaffolds provide desirable biomechanical, biochemical, and cell biological properties and are comparable to commercially available porcine reference products.

The longitudinal collagen fiber alignment of new scaffold material is supposed to imitate the natural tendon structure and mechanical properties. The manufacturing process already provides very reproducible material properties. Moreover, an industrial production process has to be established to meet regulatory requirements for medical devices. Following,* in vitro* tests focusing on regenerative processes using tenocytes or stem cells are of great interest. Animal tests have to be carried out to prove the functionality and in vivo biocompatibility of the collagen scaffolds. We expect the bovine collagen scaffolds to have beneficial effects on tendon defect healing in future clinical applications.

## Figures and Tables

**Table 1:** *Mechanical properties of the tested scaffold materials.*Ultimate load: Fmax, ultimate stress: Rm; stiffness: S; elastic modulus: EM. Data are means ± SD. For statistical analysis one-way ANOVA with Posthoc Bonferroni was conducted, ^*∗*^*p* < 0.05 compared to Test Material 1, ^*∗∗*^*p* < 0.05 compared to Test Material 2, and °*p* < 0.05 DX Reinforcement compared to Conexa™ 100.

Scaffold	Fmax (N)	Rm (N/mm^2^)	S (N/mm)	EM (MPa)
DX Reinforcement	209.7 ± 23.7^*∗* *∗∗*^	7.4 ± 0.7^*∗* *∗∗*^	24.3 ± 4.5^*∗* *∗∗* °^	20.8 ± 5.1^*∗* *∗∗*^
Conexa 100	238.9 ± 26.6^*∗* *∗∗*^	10.1 ± 0.9^*∗*^	44.4 ± 1.9^*∗* *∗∗*^	42.8 ± 3.4^*∗*^
Test Material 1	362.0 ± 32.3	21.0 ± 1.6	71.4 ± 14.7	86.9 ± 19.0
Test Material 2	309.4 ± 24.9	12.5 ± 1.4^*∗*^	73.1 ± 4.5	61.5 ± 4.0^*∗*^
